# Predicting Timelines in Healthcare Infrastructure Projects: Learnings From an Apex Tertiary Care Teaching Hospital in North India

**DOI:** 10.7759/cureus.24684

**Published:** 2022-05-03

**Authors:** Prakash S, Angel Rajan Singh, Sidhartha Satpathy, Jean Patrice Khoshbin

**Affiliations:** 1 Department of Hospital Administration, All India Institute of Medical Sciences, New Delhi, IND; 2 Office of Space and Facilities Management (OSFM), National Cancer Institute, Rockville, USA

**Keywords:** statutory requirements for hospital constructions, time overrun, healthcare project management, planning, infrastructure

## Abstract

Aim

Healthcare infrastructure projects are a requirement for the progress of the country. The aim of this study was to identify major completed healthcare infrastructure projects in a tertiary teaching hospital in Northern India and to find out the various factors that influenced the success or failures and the cost and time overrun during the project implementation.

Materials and methods

Periodical review meetings were conducted, right from the planning to the execution and commissioning of these projects. All these had been documented as minutes of the meetings, and the records of the same had been maintained. The study comprised of studying all these documents in detail and finding an answer to the research questions.

Results

Four major completed projects of a tertiary medical institute of India, All India Institute of Medical Sciences (AIIMS), New Delhi, India, were studied. These were the new Outpatient Department (OPD) Block, Burns and Plastic Surgery Block (BPS), Maternal and Child Health Block (MCH), and National Cancer Institute (NCI). Our study revealed that there was no dearth of funds, and hence, there was no cost overrun in any of the projects. Whenever the funds had to be reworked, the funds that were asked for were released. However, there was a considerable time overrun in all the projects ranging from about one to four years. The various reasons that could be attributed to this are the delay in obtaining statutory clearances, political interference, communication hurdles, improper planning, introducing a project officer late into the project, safety concerns, and the COVID-19 pandemic.

Conclusions

This study focuses mainly on the very important hurdles that were faced during the implementation of the projects and tries to suggest an average time frame for various activities for project implementation in a healthcare project in the Indian scenario. This can be taken as blueprints while planning newer healthcare projects of this magnitude.

## Introduction

“Intellectuals solve problems; geniuses prevent them.” - Albert Einstein

The economic growth of any country is primarily dependent on the development of its infrastructure, and India is no exception. In the past, it was poised to grow at 7.5% in 2019 and 7.7% in 2020 as per the International Monetary Fund (IMF). The projected real GDP of 2021 as per the IMF is 9.5% [1]. With investments envisaged amounting to 500 lakh million Indian Rupees (INR) ($1 is approximately 77 INR), there has been a considerable impetus to effectively deliver projects and programs in the country. As per the 2017-2018 Economic Survey, India will require investments of around $4.5 trillion by 2040 to develop the infrastructure for sustaining its economic growth [2].

Project discipline is country agnostic; that is, the structured project management practices of project delivery are crucial regardless of geographic location. However, challenges remain in terms of maturity in the adoption of these global best practices, the complexity of projects, and the skilled workforce.

Oxford dictionary defines infrastructure as the basic systems and services that are necessary for a country or an organization to run smoothly, e.g., buildings, transport, and water and power supplies. These are usually carried out in project mode. A project is defined as “a temporary endeavor undertaken to create a unique product, service, or result [3].”

Proper planning and execution of these projects is an important requirement, failing which progress becomes tardy. A study on project management will be helpful in identifying mistakes done in earlier projects. Lessons learned from this will enable us to take remedial actions so that future projects do not suffer.

In defining project management success, the focus is on the project management process and on the successful accomplishment of the project, i.e., time, budget, and quality. The project product success focuses on the product or service, which is the outcome of the project.

To serve the exceptional infrastructure growth and address the associated challenges, the Central Government of India has launched several initiatives. Such initiatives have been successful to an extent in easing out some of the core issues such as land acquisition and regulatory approvals. In addition, The National Institute for Transforming India (NITI) Aayog (the premier think tank of the Government of India, providing directional and policy inputs) has recommended implementing model agreements for improving contractor procurement, which advocates adopting engineering, procurement, and construction (EPC) contracting, incentivizing contractors for early project completion, and stage payments that can potentially align the interest of both clients and contractors to the project and reduce the number of claims [1].

The business of building infrastructure is one that involves long gestation periods and is often closely intertwined with public opinions impacting the project outcomes. Given this backdrop, it is important to focus on the core issues and the opportunity that lies therein, because the road to growth cannot happen without putting in place the infrastructure backbone, which then has a multiplier effect on which way the economy and we go as a nation.

Additionally, the infrastructure development in India is prone to dependency on external factors (land, environment, socioeconomic, community, regulatory, market forces, etc.) and has a fair set of challenges. Added to that are the sectorial nuances, with project types that are geographically spread/linear and those that are area-based. While there may have been a few success stories, as a whole, our performance falls short significantly and on a consistent basis [1].

The Ministry of Health and Family Welfare has also given importance to the requirement and improvement of healthcare infrastructure. Excerpts from their annual report for 2020-2021 read, “The approach is to increase access to the decentralized public health system by establishing new infrastructure in deficient areas and upgrading the infrastructure in the existing institutions. There is also a need to strengthen the role of the public sector in social protection against the rising costs of healthcare and the need to provide a comprehensive package of services without reducing the prioritization given to women and children’s health. There has been a significant improvement in the creation of new facilities and infrastructure, though adequate human resource at these facilities by qualified health personnel, still remains a challenge. Availability of drugs has improved at all levels and the robust logistic arrangement for procurement and storage of these drugs has been put in place.” [4].

Very few studies have concentrated on the project management of healthcare projects. One such research found various factors that cause delays in hospital projects in Vietnam. Factor analysis uncovered six main principle factors: the ability of the owner and contractor, the ability of the consultant and contractor, outside effects, the ability of designers, the ability of supervisors, and other causes. The study not only found the principal factors but also ranked the factors under a combined view and others’ views. Under the combined view, the first cause is “the finance of the owners.” The top five causes of delay in hospital projects included financial difficulties of the owner, lack of supervisor responsibilities, change of design by the owner, incompetence of the contractor, and inadequate contractor experience. The authors analyzed the Spearman coefficient of the group views together and proved that they have no relationship with each viewpoint [5].

The COVID-19 pandemic changed how we looked at project management for healthcare projects. Newer hospitals, even if it means by makeshift arrangements, came in with great speed. The mantra was to come up with more beds in an easier and a robust way. We have had many examples from countries such as China and Kazakhstan that build hospitals in about 10 days’ time. In our country, the Defence Research and Development Organisation came up with a thousand bedded makeshift hospital in 12 days’ time, and the hospital by the Government of the National Capital Territory (NCT) of Delhi was built for 10,000 patients in record time. All India Institute of Medical Sciences (AIIMS) on its own converted the AIIMS Trauma Center, the National Cancer Center, and the Private Wards in AIIMS to COVID-19 wards on a fast track basis. AIIMS also came up with a pediatric casualty within 72 hours [6].

Rationale for this study

All India Institute of Medical Sciences (AIIMS) has always reinforced its position as one of the best hospitals in India, on account of having the best doctors, along with excellent infrastructural and research facilities for various diseases. The institute, which was established in 1956, is also a leading training institute for medical students. Medical science here is highly advanced, which is the reason why AIIMS as a hospital attracts so many patients from different corners of the world. It is globally renowned and recognized for its technology-driven approach.

This hospital is thus facing increasing demand of patients both in the outpatient department (OPD) and inpatient. To cater to this, various new projects had come up in the past, and there are new master plans that are being deliberated upon. This is to ease the congestion of the main hospital and at the same time provide quality and yeoman services to the patients coming from all parts of India and abroad. These projects provide a perfect milieu to look into the various hassles that a project manager could face.

AIIMS, New Delhi, in its seventh decade of existence, has embarked on a journey of massive expansion and upgrade of its existing facilities. AIIMS, New Delhi, is underway a mammoth 150,000 million INR masterplan project that will change the way the hospital functions by incorporating newer technologies, improving hospital layouts, and making it more patient- and staff-friendly. AIIMS is all set to reinvent itself as a world-class medical university with an enhanced focus on research, academics, and quality campus life.

Hence, we planned to study the various reasons for the delays and other factors that affected the project implementation of various major infrastructure projects at AIIMS. The learnings from this study could go a long way in mitigating various issues that could crop up during the implementation of this mammoth new expansion project.

## Materials and methods

The present study aims to ascertain the major hospital infrastructure projects of AIIMS, New Delhi, costing over 1000 million INR and commissioned within the past 20 years, to document the challenges faced during the implementation of the aforementioned projects of AIIMS, New Delhi, and to measure the project achievements of these healthcare infrastructure projects of AIIMS, New Delhi.

The study is a qualitative type of study. Various records are available in the Engineering Services Division, and the project offices of various projects, such as the Standing Finance Committee (SFC) Reports, Expenditure Finance Committee (EFC) memorandum, and Cabinet Committee on Economic Affairs (CCEA) note records, including the project office records, were studied in detail. The available records in the Finance and Accounts Division were also studied in detail. These helped in documenting the major hospital infrastructure projects of AIIMS costing over 1000 million INR and commissioned in the last 20 years.

Available records regarding the planning and execution of the completed and ongoing projects were studied in detail. The various Project Committee meeting reports and Core Committee meeting reports and minutes of the meetings with ministries were studied and analyzed in detail to learn about the various hurdles faced at all the steps of project management. Unstructured interviews were conducted with the major stakeholders to find the challenges faced and how they were tackled for the successful commissioning of the projects. The interviews were conducted with four project officers of each of the projects, four consultants of these projects Hospital Services Consultancy Corporation (HSCC) (India) Limited, and the resident administrators who helped the project officers supervise the project. There were a total of 23 duty officers who played an instrumental role during the course of the implementation of these projects, and all of them were interviewed. Timelines from tender documents and deadlines/targets were obtained from the minutes of meetings. Relevant available data were collected to measure the project achievements of these hospital projects.

## Results

After going through various records, the four projects that come within the ambit of our study among the major projects of AIIMS are the Burns and Plastic Surgery Block (BPS), Jai Prakash Narayan Apex Trauma Center (JPNATC) Campus, AIIMS, New Delhi; Maternal and Child Health Block (MCH), Masjid Moth Campus, AIIMS, New Delhi; New Rajkumari Amrit Kaur (New RAK) OPD, Masjid Moth Campus, AIIMS, New Delhi; and National Cancer Institute (NCI), AIIMS, Jhajjar, Haryana. The costs incurred for these projects are shown in Table 1.

**Table 1:** Costs incurred on the projects

Projects	Cost (in million INR)
Burns and Plastic Surgery Block (BPS)	2478.5
Maternal and Child Health Block (MCH)	7258.4
New Rajkumari Amrit Kaur OPD (New RAK)	5729.9
National Cancer Institute (NCI)	20,350

The various challenges that were seen during the implementation of the projects are summarized in Table 2.

**Table 2:** Summary of various hurdles faced during the implementation of the projects

Phase	Project stage	Hurdles
Prerequisites	Finances	Finances were never a hurdle for the projects. In fact, good meticulous planning had been done for the planning of all the projects, and there was never any delay in the release of funds. The only issues that have been highlighted were that in some cases, the consultant party had delayed the handing over of money to the contractors, which had to be tackled.
	Site selection	This was a major hurdle in two of the projects, i.e., the National Cancer Institute (NCI) and the Burns and Plastic Surgery Block. Also, political interference played an important role in the execution of manpower of the projects.
	Statutory requirements	Most of the delays anticipated were because of the delay in obtaining many of the statutory clearances from various bodies, such as the Archaeological Survey of India (ASI), Environmental Impact Assessment (EIA) clearance, Director General Civil Aviation (DGCA) clearance, and water and electricity clearances.
Planning	Planning	When we go through the observations, it is noticed that almost all the hurdles faced during the construction phase were because most of the planning was poorly done, without taking all the stakeholders into confidence. This led to most of the changes in plans, and hence, there were fines that were levied.
	Human resources	Human resources were not planned properly as at times there were no proper recruitment rules framed, or they were framed arbitrarily without taking into consideration the number of staff for similar posts in various other hospitals or without a proper framework or norms.
	Project officer	Qualified project officers were brought in the middle of the projects. The study of these projects has thrown light on the importance of such a person to steer the projects in the right direction. Without one such person unifying the command, coordinating it, and providing direction, projects were going haywire under multiple leadership.
Execution	Safety measures	Incidents of accidents that led to litigations and compensations were noted, which also led to time lost in inquiries.
	Regular supervision and communication with the stakeholders	Weekly follow-up, monthly meetings with the stakeholders, quarterly meetings at the institute levels, and yearly supervision by the government were some of the good salient features of the project execution.
	COVID-19 pandemic	The COVID-19 pandemic played a dual role in the project implementation. Although at one stage it did halt the progress of the projects because of the nonavailability of manpower and materials, at the same time, there was immense pressure from the government to complete and start these hospitals on a war footing as these would add further beds to the country during the pandemic. The bureaucracy did help by giving concessions in the lockdown for faster execution of the projects.

Table 3 summarizes the period required for these projects.

**Table 3:** Area and number of floors vis-à-vis the time taken for construction G: ground floor, B: basement, BPS: Burns and Plastic Surgery Block, MCH: Maternal and Child Health Block, New RAK OPD: New Rajkumari Amrit Kaur Outpatient Department (Dr. Rajkumari Amrit Kaur was the first Health Minister of India), NCI: National Cancer Institute

Block/center	Floors	Area	Total period of construction (proposed)	Actual period of construction
BPS	G+3B+8	24,027 m^2^	24 months	36 months
MCH	G+3B+8	45,352 m^2^	24 months	73 months
New RAK OPD	G+3B+8	93,352 m^2^	40 months	56 months
NCI	Most of the buildings have G+2B+5 floors	252,000 m^2^ (which includes 100,207 m^2^ of residential dwellings)	45 months	60 months

**Figure 1:**
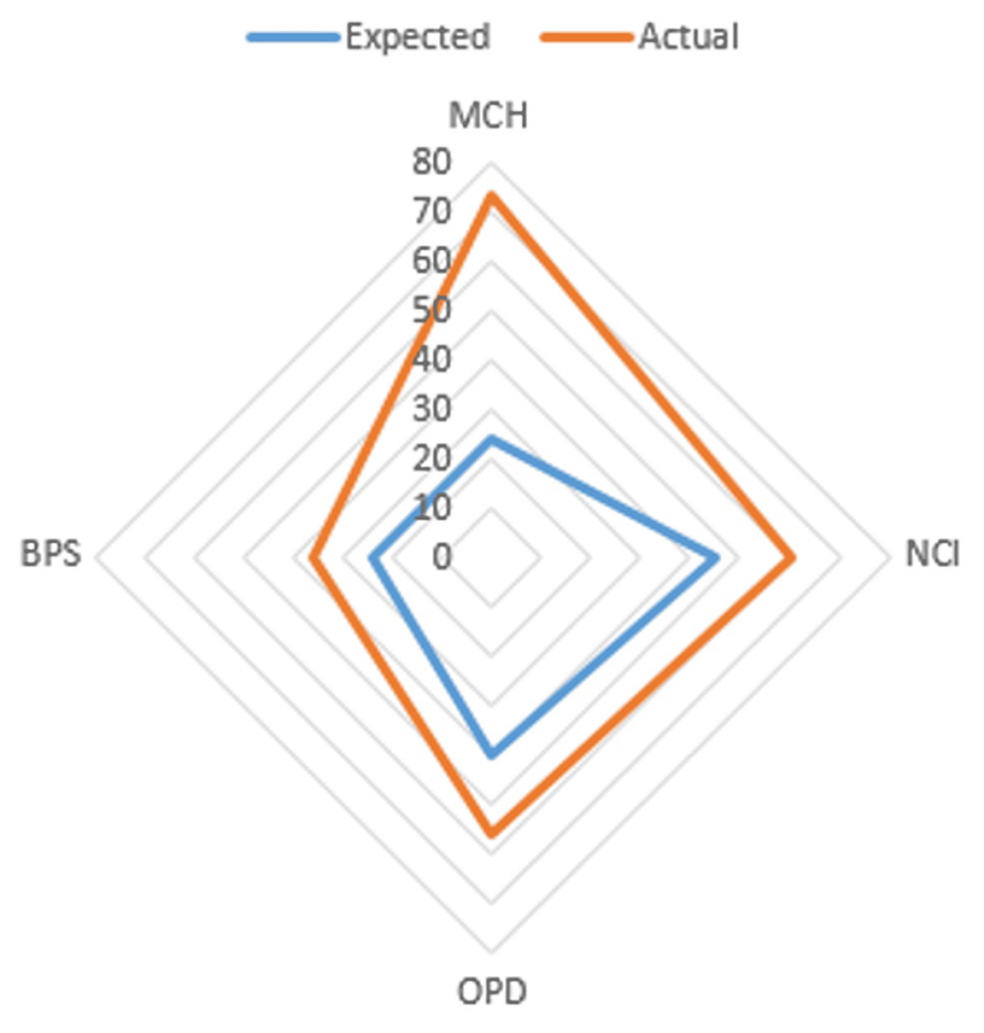
Comparison between the expected and the actual period of the projects (in months) BPS: Burns and Plastic Surgery Block, MCH: Maternal and Child Health Block, New RAK OPD: New Rajkumari Amrit Kaur Outpatient Department (Dr. Rajkumari Amrit Kaur was the first Health Minister of India), NCI: National Cancer Institute

When plotted graphically, it is seen that all the projects had time overrun (Figure 1).

At the end of every year, a yearly progress meeting is held under the chairmanship of the representative of the Prime Minister’s Office. This meeting is attended by all the stakeholders of the projects, such as the planners, users, project officers, consultants, contractors, and the engineering team. The meeting is also attended by the administrators, including those from the Finance and Accounts wing. An important part of the meeting is that it is attended by senior echelons of the government from various ministries such as health and finance. The progress of each project is deliberated upon vis-à-vis the actual planned timelines. The completed percentage of the entire project is also measured. If required, newer timelines are drawn. Also, such meetings help in the intersectoral coordination and help in the removal of hurdles by different departments or agencies and by brainstorming and discussions with all concerned.

**Figure 2:**
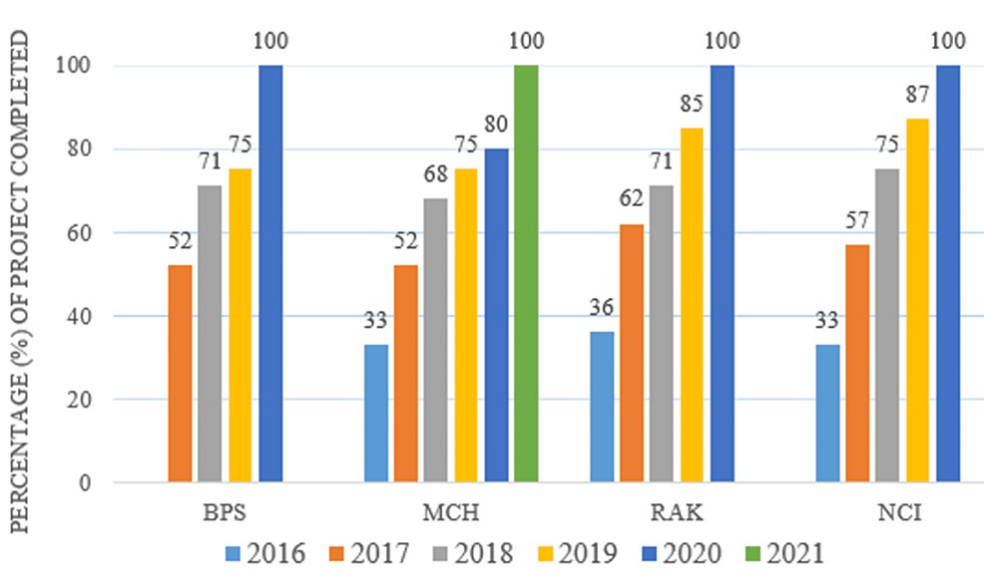
Timeline chart showing the progress of the pace of work (work completed in percentage at the end of the year) for the basic infrastructure (data collected from the yearly progress meeting presented to the Prime Minister’s Office) BPS: Burns and Plastic Surgery Block, MCH: Maternal and Child Health Block, RAK: New Rajkumari Amrit Kaur Outpatient Department, NCI: National Cancer Institute

The timeline chart showing the progress of the pace of work (work completed in percentage at the end of the year) for the basic infrastructure is shown in Figure 2 (data collected from the yearly progress meeting presented in the Prime Minister’s Office).

The average period of milestones for different activities (expected vis-à-vis actual) is summarized in Table 4.

**Table 4:** Average period of milestones for different activities (expected vis-à-vis actual) and the delay

Milestone	Duration of activity (planned (average))	Average time (actual)	Average delay
Concept design and development	1 month	3 months	2 months
Approval of concept	1 month	1 month	Nil
Submission for Environment Impact Assessment (EIA) clearances	1 month	2 months	1 month
Detailed preparation of drawings – architectural, structural, electrical, plumbing, services, landscaping, and elevators	3 months	3 months	Nil
EIA clearances	3 months	20 months	17 months
Height clearance	1 month	3 months	2 months
Pollution control board clearance	1 month	2 months	1 month
Clearance from Green Tribunal	1 month	1 month	Nil
Water requirement	1 month	6 months	5 months
Electricity requirement	1 month	8 months and permanent meter obtained after handing over	7 months
Archaeological Survey of India clearance	15 days	2 months	45 days
Submission and drawing of approval from local bodies and the Atomic Energy Regulatory Body (AERB) and Geotechnical Survey (soil testing)	2 months	12 months	10 months
Preparation of tender documents	2 months	6 months	4 months
Tendering and award of work	3 months	8 months	5 months
Actual construction	3200 m^2^/month	1800 m^2^/month	
Equipment	During the process of construction	During the process of construction	
Furniture	6 months	12 months	6 months
Manpower	12 months	36 months	24 months
IT work	6 months	12 months	6 months
Handing over	1 month	1 month	Nil
Fire clearance	At the start, in between, and in the end	Approximately takes 15 days if no omissions are found	

## Discussion

The creation of high-quality and durable infrastructure is required for rapid economic development and requires a sustained investment that is supported well by technological innovation, a skilled workforce, and good project management. Healthcare, hence, cannot distance itself from such a concept. In its endeavors to provide excellent healthcare in the most important health institute of the country, the Government, too, has embarked on the journey of approving various projects.

Delay in obtaining clearances and frequent changes of plans/poor planning were the most important reasons for the time overrun.

Environmental Impact Assessment (EIA) was one big hurdle in almost all the projects. The files for the same were returned back for clarifications and discussions multiple times, as all the aspects for the protection of the environment had to be taken care of. The intention behind the Environmental Impact Assessment is to “streamline the processes” of the grant of environment clearance while balancing the principle of “sustainable development” and the government’s policy of “ease of doing business.” Hence, a detailed study of this requirement and meeting all the clauses in the requirement are mandatory for such big projects.

These are the various deficiencies of the system. Most of the time, the delay has been in obtaining all clearances from different authorities. None of the clearances including the timelines for various meetings were honored. The reasons for the same are manifold: faults in documents (preparing them without appropriate consideration for the fees and penalties), delay in getting an appointment from the authorities for inspections, changes in rules and regulations such as the introduction of GST, and inappropriate marking of vouchers (e.g., the vouchers were made in the name of Hospital Services Consultancy Corporation (the consulting firm of the projects) and not in the name of the director of AIIMS, which did not allow customs fee relaxations).

Political interference has also caused some issues, such as delays in finalizing the documents, especially for tendering of manpower. The same has been reflected in many studies [7-9].

The fall of the Tower of Babel is a well-known example of communication blocks. This is wherein a project officer, too, can play an important role. In many of the cases that we have studied, it was the project officer who ensured that all the stakeholders think in one direction, and this has helped in the solving of major issues of the projects. We need to remember that, as shown by various studies such as that conducted by Pauget et al. [10] and Zulch et al. [11], it was communication that has helped in the successful completion of complex hospital projects.

Planning has affected almost all our projects understudy as the concept of project management has not been used in the true spirit. A project officer is required to coordinate among various departments so that all the various departments work in tandem. Many of the structural changes and further the levying of fines by regulatory bodies were because of the unplanning and the unlocking of the projects at an earlier stage without detailed discussions with the stakeholders. The same could have been prevented as echoed in various studies, especially by Asian authors such as Devi [12] and Giri [13]. The various mitigation factors available in the literature to prevent time and cost overrun could not be implemented [14-16]. This is because, in most cases, a planner, the project officer, was brought in the middle of the projects. Hierarchy was one important hurdle that the projects faced. Since there were many specialists from many fields and different departments involved, each department wanted to create an important impression and get bigger space and bigger offices for their departments. There were many leaders overseeing the projects, but none steered it in one direction. Once the project officer was introduced, the projects started moving at a good pace and could be completed satisfactorily.

Engineers and architects would think and plan as per their domain. However, their planning has to be steered by a project officer, especially a specialist in this field, and allow the engineer to think in sync with the rest of the people in the project. This will also help in planning for a good hospital infrastructure from the infection control and safety point of view and prevent future ailments such as sick building syndrome in an already sick patient [17,18]. Thus, an integrated project delivery system can help in improving projects of these magnitudes.

Safety during construction is another important mandate that should find its way into our projects. Two of the four projects studied did have accidents that have resulted in the levying of compensations. Also, there were objections by environmentalists and nearby residents because of due care not being taken and also because the local laws in relation to the state were not being followed. Authors such as Zahoor et al. [19] and Haslam et al. [20] have highlighted the requirement of safety measures in their studies.

According to the Ministry of Statistics and Programme Implementation (MoSPI) project database, as of January 2018, 345 projects have incurred a cost overrun of 21.9 lakh million INR, and 354 projects have an average delay of 45 months [1]. As per our study, there has been an average delay of 23 months with three of the projects in the range of 12-15 months and the MCH block having a delay of 49 months. This was because many of the hurdles were not kept in mind while planning these projects.

The COVID-19 pandemic has played an important role in the delivery of the projects. As mentioned before and supported by various studies, political influence has an effect on project delivery. At one point, the entire world was grappling for beds to take care of the increasing demand of positive cases, and AIIMS had many infrastructure elements that were in the early phase of completion. Hence, although there were many issues related to COVID-19 regarding the nonavailability of laborers and materials [16], still since there was political pressure to complete these projects on time, so as to make them open for COVID-19 patients, there was considerable help received from the bureaucracy. This was a classical example of the theory of punctuated equilibrium.

The limitation of the study is that although there were many projects that have been developed at this institute, we restricted ourselves only to those that were above 1000 million INR and those that were commissioned and completed in the last 20 years. We have also not quantified or given a relative ranking to the problems that were the causes of the project overrun.

## Conclusions

Many extrinsic factors, such as mandatory statutory clearances and political interferences, and intrinsic factors, such as poor planning and the absence of a project officer from the beginning, can affect the progress of healthcare infrastructure projects. The need of the hour is to have robust planning for these projects, regular supervision, and strategic management to highlight and solve complex issues that may crop up at any time. Various evaluation of the progress of the projects and the removal of hurdles are required. It is only then that we can cut down the factors that affect projects, especially time and cost overrun, and thus have value for these projects and at the same time maintain the quality.
